# Effects of DC Magnetic Fields on Magnetoliposomes

**DOI:** 10.3389/fmolb.2021.703417

**Published:** 2021-09-13

**Authors:** L. Nuñez-Magos, J. Lira-Escobedo, R. Rodríguez-López, M. Muñoz-Navia, F. Castillo-Rivera, P. X. Viveros-Méndez, E. Araujo, A. Encinas, S. A. Saucedo-Anaya, S. Aranda-Espinoza

**Affiliations:** ^1^ Laboratory of Biophysics and Soft Matter, Instituto de Física, Universidad Autónoma de San Luis Potosí, San Luis Potosí, Mexico; ^2^ Ingeniería en Nanotecnología, Universidad de La Ciénega del Estado de Michoacán de Ocampo, Sahuayo, Mexico; ^3^ CONACyT–Instituto de Geología de la Universidad Autónoma de San Luis Potosí, San Luis Potosí, Mexico; ^4^ Unidad Académica de Ciencia y Tecnología de la Luz y la Materia, Universidad Autónoma de Zacatecas, Zacatecas, Mexico; ^5^ Departamento de Matematicas y Física, Instituto Tecnológico y de Estudios Superiores de Occidente, San Pedro Tlaquepaque, Mexico; ^6^ Laboratory of Magnetism, División de Materiales Avanzados, Instituto Potosino de Investigación Científica y Tecnológica, San Luis Potosí, Mexico; ^7^ Unidad Académica de Estudios Nucleares, Universidad Autónoma de Zacatecas, Zacatecas, Mexico

**Keywords:** magnetoliposomes, liposome deformation, magnetic nanoparticles, lipid membranes, magnetic field, biological effects

## Abstract

The potential use of magnetic nanoparticles (MNPs) in biomedicine as magnetic resonance, drug delivery, imagenology, hyperthermia, biosensors, and biological separation has been studied in different laboratories. One of the challenges on MNP elaboration for biological applications is the size, biocompatibility, heat efficiency, stabilization in physiological conditions, and surface coating. Magnetoliposome (ML), a lipid bilayer of phospholipids encapsulating MNPs, is a system used to reduce toxicity. Encapsulated MNPs can be used as a potential drug and a gene delivery system, and in the presence of magnetic fields, MLs can be accumulated in a target tissue by a strong gradient magnetic field. Here, we present a study of the effects of DC magnetic fields on encapsulated MNPs inside liposomes. Despite their widespread applications in biotechnology and environmental, biomedical, and materials science, the effects of magnetic fields on MLs are unclear. We use a modified coprecipitation method to synthesize superparamagnetic nanoparticles (SNPs) in aqueous solutions. The SNPs are encapsulated inside phospholipid liposomes to study the interaction between phospholipids and SNPs. Material characterization of SNPs reveals round-shaped nanoparticles with an average size of 12 nm, mainly magnetite. MLs were prepared by the rehydration method. After formation, we found two types of MLs: one type is tense with SNPs encapsulated and the other is a floppy vesicle that does not show the presence of SNPs. To study the response of MLs to an applied DC magnetic field, we used a homemade chamber. Digitalized images show encapsulated SNPs assembled in chain formation when a DC magnetic field is applied. When the magnetic field is switched off, it completely disperses SNPs. Floppy MLs deform along the direction of the external applied magnetic field. Solving the relevant magnetostatic equations, we present a theoretical model to explain the ML deformations by analyzing the forces exerted by the magnetic field over the surface of the spheroidal liposome. Tangential magnetic forces acting on the ML surface result in a press force deforming MLs. The type of deformations will depend on the magnetic properties of the mediums inside and outside the MLs. The model predicts a coexistence region of oblate–prolate deformation in the zone where *χ* = 1. We can understand the chain formation in terms of a dipole–dipole interaction of SNP.

## 1 Introduction

Magnetic nanoparticles (MNPs) have widespread applications in biotechnology and environmental, biomedical, and materials science (Singamaneni et al., 2011; Akbarzadeh et al., 2012; Kim et al., 2013; Bohara et al., 2016). MNPs have potential use in biomedical applications such as cell labeling (Kolosnjaj-Tabi et al., 2013; Min et al., 2011; Wilhelm and Gazeau, 2008), magnetic hyperthermia (Hedayatnasab et al., 2017; Abenojar et al., 2016; Laurent et al., 2011), drug delivery (Kost et al., 1987; Tietze et al., 2015; McBain et al., 2008; Huang et al., 2016), magnetic resonance imaging (Chaughule et al., 2012; Huang et al., 2010; Zhu et al., 2017), magnetic separation (Mandel and Hutter, 2012; Stephens et al., 2012; Luo and Nguyen, 2017), and enrichment of DNA (Xie et al., 2007; Zhou et al., 2013; Min et al., 2014; Arakaki et al., 2008). The main characteristics of MNPs are their subcellular size, ranging from a few nanometers to tens of nanometers, allowing them to interact with nano-molecular-sized biomolecules. Functionalization of MNPs to target specific molecules is one of the main challenges (Akbarzadeh et al., 2012; Kim et al., 2013; Bohara et al., 2016). Magnetoliposome (ML), a lipid bilayer of phospholipids encapsulating MNPs, is a system that is used to reduce toxicity while also being biocompatible and biodegradable (Sakuragi et al., 2017; Alonso et al., 2016; Kong et al., 2014). The design of MLs is a spontaneous process that effectively encapsulates MNPs in a lipid bilayer (De Cuyper and Joniau, 1988). In the work of Bain et al. (2015), they used the electroporation method where polymersomes are suspended in an iron solution. By controlling the electroporation parameters, they control iron flow to manipulate MNP sizes. With this methodology, they crystalize MNPs within the polymersome. Bakhshi et al. (2016) used a combination of electrohydrodynamic atomization and electroporation to produce MLs with near-monodisperse magnetite inside the liposome. ML shielding prevents lysosomal degradation by avoiding decomposition of MNPs (Soenen et al., 2009). MLs are stable in organic solutions, and the use of this system for biomedical applications has exponentially grown over the past few years. The manipulation of MNPs by external magnetic fields is the primary interest of this study. Under the DC magnetic field, MNPs auto-arrange into 3D linear chains due to strong dipolar interaction. Encapsulated MNPs can be used as a potential drug and a gene delivery system (Kost et al., 1987; Fortin-Ripoche et al., 2006). In the presence of magnetic fields, MLs can be accumulated in a target tissue by a strong gradient magnetic field (Pradhan et al., 2010).

The present work studies the effects of DC magnetic fields on MLs with encapsulated SNP at different concentrations. We found that the encapsulated SNP tends to form chain formations under the influence of a DC magnetic field without ML deformation. The size and distribution of SNP chains inside the MLs depend only on the concentration of nanoparticles. When SNP accumulates in the membrane interface, MLs deform in the direction of the applied DC magnetic field, and they attain a prolate deformation without chain formation. The chain SNP formation and ML deformation were observed by phase-contrast microscopy. We propose a model to explain ML deformations based on the energy analysis applied by the magnetic field over the MLs. Dipole–dipole SNP interactions can explain the chain formation (Myrovali et al., 2016). Magnetometry hysteresis loop measurements show the superparamagnetic nature of the particles. Here, we want to bring attention to the possibilities offered by uniform magnetic fields for the assembly and manipulation of encapsulated SNPs in liposomes.

## 2 Experimental Section

### 2.1 Materials

1,2-Dioleoyl-sn-glycero-3-phosphocholine (DOPC) was purchased from Avanti Polar Lipids (Alabaster, AL). Sodium citrate dihydrate (Na_3_C_3_H_5_O(COO)_3_ ⋅2H_2_O), sodium hydroxide (NaOH), nitric acid (HNO_3_), ammonium hydroxide solution (NH_4_OH), ferric chloride (FeCl_3_ ⋅ 6*H*_2_ *O*), ferrous chloride (FeCl_2_ ⋅ 4*H*_2_ *O*), and chemicals and solvents used to rehydrate MLs were purchased from Sigma Chemicals (St. Louis, MO). Sucrose and glucose were purchased from B. Braun Medicals Inc. (Irvine, CA). Rectangular NdFeB permanent magnets and steel rods were purchased from La Paloma (Mexico City).

### 2.2 Synthesis of Superparamagnetic Nanoparticles

Superparamegnetic nanoparticles were synthesized by coprecipitation of ferrous and ferric salts in alkaline and acidic aqueous solutions (Tokarev et al., 2015; Lu et al., 2007). With a stoichiometric ratio of 2Fe^3+^: Fe^2+^, 4.43 g FeCl_3_ ⋅ 6H_2_O and 1.625 g of FeCl_2_ ⋅ 4H_2_O were dissolved in 190 ml deionized water at room temperature by magnetic stirring in a beaker.

Under vigorous stirring, 10 ml of ammonia was dropped to the beaker at a constant speed using a burette. Immediately, a black precipitate is formed. After stirring for 10 minutes, the obtained precipitate were magnetically separated from the solution with the aid of a magnet and washed twice with water until we get a pH value of 7.0.

To disperse the particles in water, they were surface-complexed with citrate ions employing the following process: first, the surface electric charges of the particles were converted from negative to positive by washing twice with 2 M HNO_3_. Then, the precipitate was washed with DI water. The pH was raised to 2.5 using NaOH. While keeping a pH of 2.5, 5 ml of sodium citrate (0.5 M) was added in drops. The solution was stirred for 120 min. Particles were separated and diluted in 100 ml of water, and the pH was raised to 6.

The concentration in *mg*/*ml* of the nanoparticles was obtained using the following protocol: an aliquot of 1 ml of solution was taken and weighed. The liquid solution was evaporated by heating at 90°C for 1 hour. The sample was dried under vacuum to constant weight. By weight differences of the resulting powder and the aliquot, the resulting concentration was 16.1 mg/ml of SNPs.

### 2.3 Magnetoliposome Formation

DOPC phospholipid at 4 mg/ml was used to prepare liposomes by the thin-film rehydration method. Lipids were dissolved in chloroform: methanol (2:1) at room temperature. Then, 12 ml of the lipid solution was deposited at the bottom of the glass bottle. The lipid solvent was evaporated under vacuum to form a thin layer of lipid over the bottom surface of the bottle. Afterward, 4 ml of magnetite solution was poured into the bottle [Three concentrations were used, 5, 3, or 1*%* (0.8 mg/ml, 0.48 mg/ml, and 0.16 mg/ml, respectively)]. Rehydration takes place in an oven at 60°C for 1 h to form multilamellar liposomes. Giant polydisperse liposomes were observed in an inverted phase-contrast microscope (Leica DMIL LED). After formation, we found two types of MLs: one type is tense with SNPs encapsulated and the other is a floppy vesicle that does not show the presence of SNPs.

### 2.4 Transmission Electron Microscopy

**FIGURE 1:**
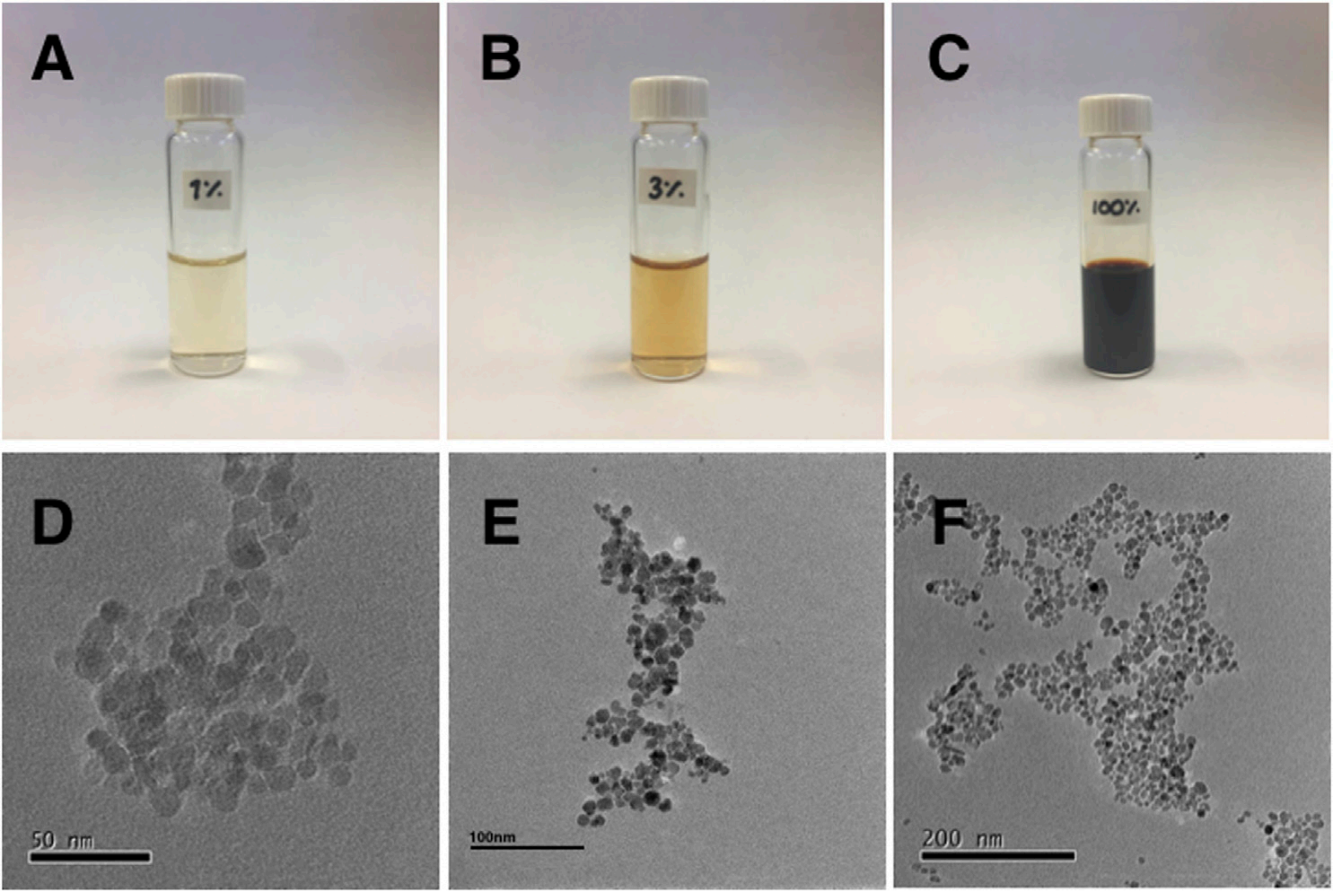
Photographs of vials containing the SNPs solutions at concentrations of **(A)** 1*%*, **(B)** 3*%*, and **(C)** the original stock solution, labeled as 100*%*. **(D–F)** TEM micrographs of the SNPs different zones of the sample with different magnifications.

MNPs were characterized using transmission electron microscopy (TEM). Drops of the suspended MNPs were deposited on a carbon film grid and vacuum-dried. Micrographs of the MNPs obtained with a JEOL2100 operated at 70 kV show the presence of apparently octahedral MNPs with 12 nm average size, Figure 1.

### 2.5 Magnetic Characterization

**FIGURE 2:**
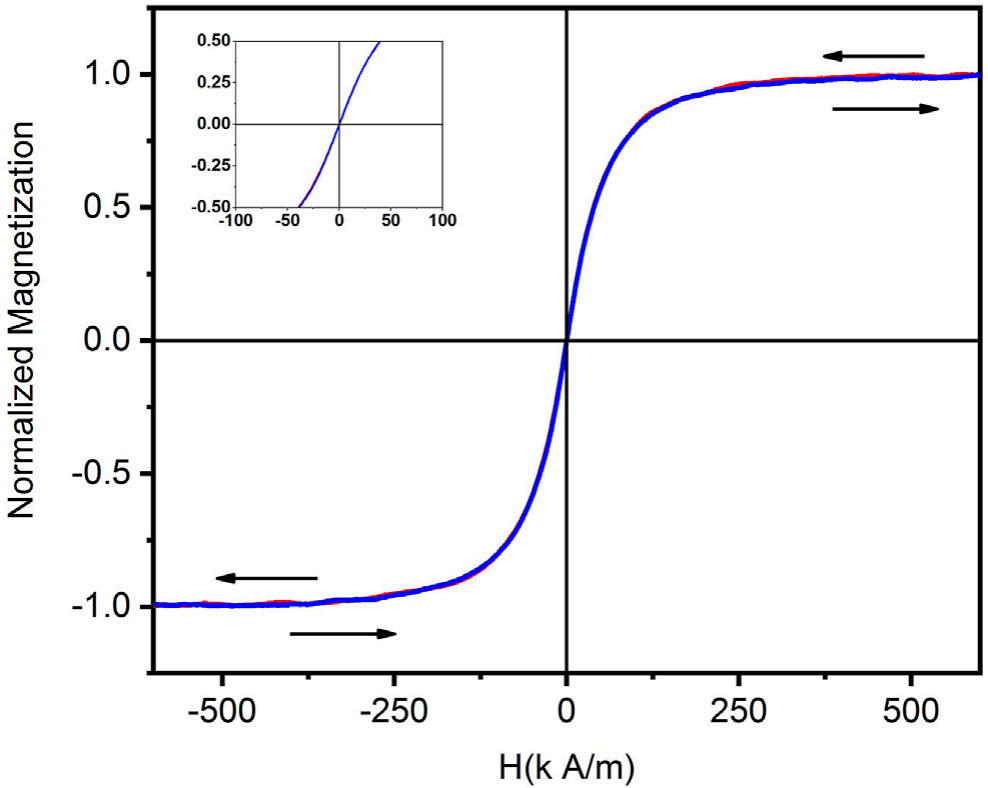
Hysteresis loop of the SNPs measured at room temperature. The inset shows a magnification near the zero field, where a null coercivity confirms the superparamagnetic behavior of the particles.

Magnetic hysteresis curves were measured at room temperature for the SNPs using an AGM MicroMag 2,900/3,900 magnetometer. The M-H curve of SNPs shows a superparamagnetic behavior (see the inset in Figure 2).

### 2.6 DC Magnetic Field Chamber

**FIGURE 3:**
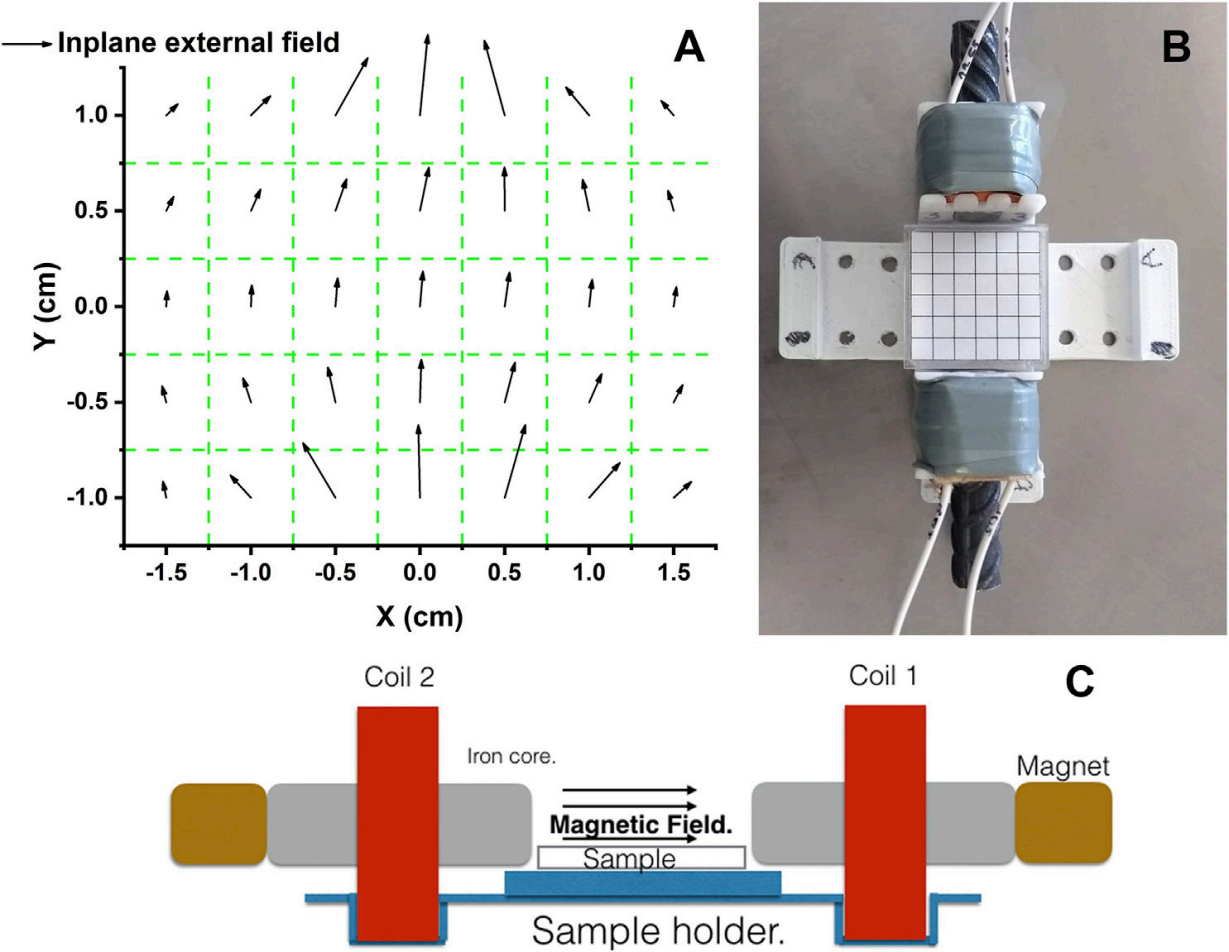
Experimental setup for the DC magnetic field chamber. Vectorial maps of the in-plane field (9̃7*%*) mapped over the working space is shown in **(A)**. **(B)** is a top view of the magnetic chamber. **(C)** is a schematic representation of a side view of the experimental set up.

A homemade magnetic chamber, see Figure 3, was used to observe MLs in real time when an external DC magnetic field was applied. The experimental chamber consists of two electromagnet coils, each with steel rods as a core. We use NdFeB magnets to change the strength of the external magnetic field. A dual benchtop power supply (Matrix MPS3005L-3) operated each coil separately. Depending on the current applied at the chamber coils, we obtain an external magnetic field of 0.0036–0.0956 T. A Gaussmeter (Lakeshore 450) was used to measure the magnetic field. Figure 3A shows the measured magnetic vector field map in the working zone. We can see that about 97*%* of the field flux along the in-plane direction and less than a $\frac{\pi }{60}$ degrees deviation from the axis along the steel rods are obtained at the center of the chamber. The separation between the steel rods cores was 3.0 cm. The working space is a 9 cm^2^ area, which corresponds to the zone with the square pattern in Figure 3B. The chamber was mounted over the phase contrast inverted microscope (Leica DIL LED). We used an objective of ×40 to observe the experiments. The images were recorded using a Leica camera mounted on the upper part of the microscope. The SNP encapsulated into the liposome’s response to DC magnetic fields was monitored under room temperature conditions and for periods no longer than 5 minutes. The magnetic field chamber gets overheated, and before another experiment takes place, we let the system cool down.

## 3 Results and Discussion

**FIGURE 4:**
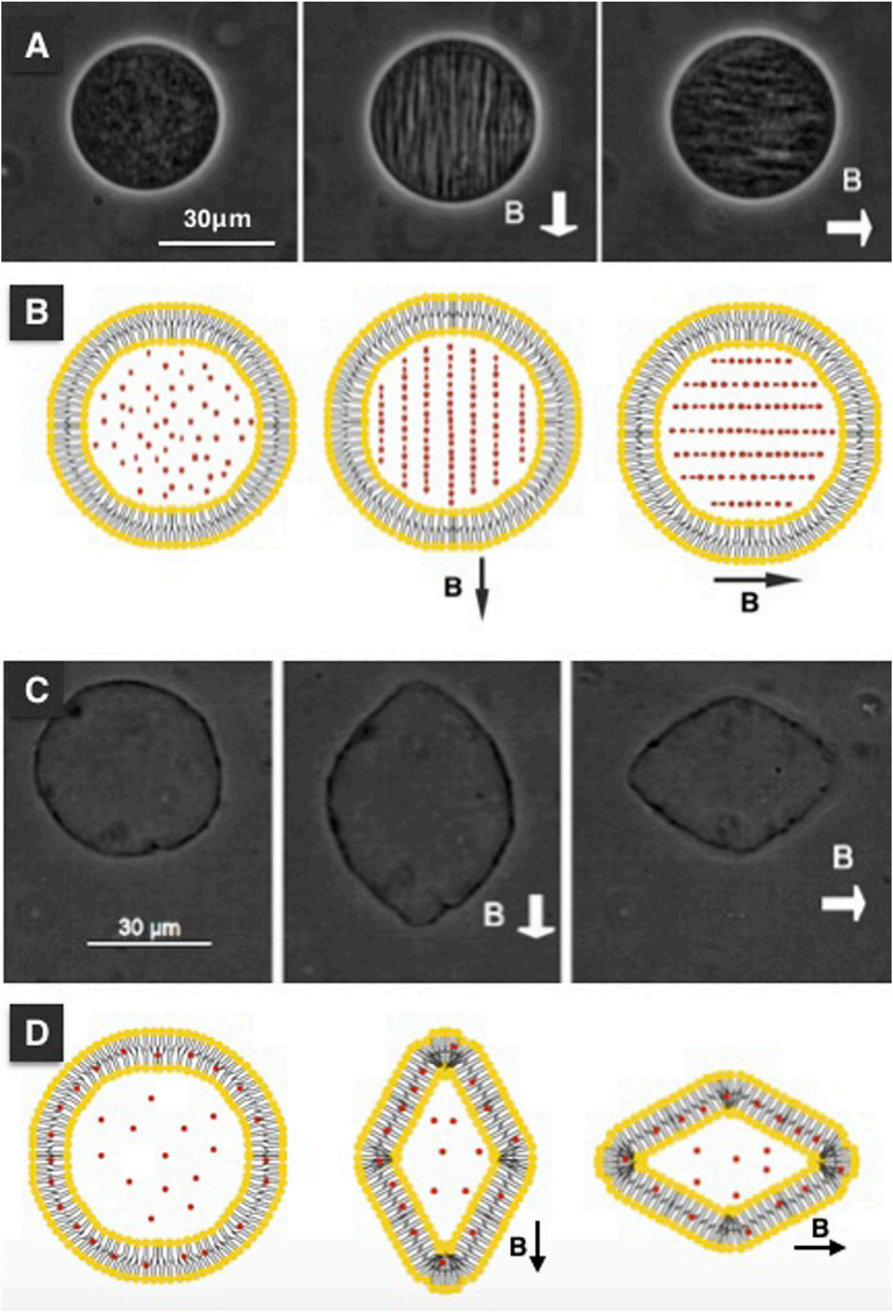
Chain formations and magnetodeformation with encapsulated SNPs. Chain formation of SNPs take place in the inner medium of the MLs, and in other cases they deform in the direction of the DC magnetic field applied. **(A)** shows the MLs when no field is applied, next image when a DC magnetic field is applied. **(B)** is a cartoon representation to illustrate the SNPs are forming chains. When rehydration takes place (in this case SNPs concentration is at 1*%*), MLs with the excess area is observed, see **(C)**. When a DC magnetic field is applied, this type of MLs deforms in the direction of the DC magnetic field. If the direction of the magnetic field is rotated by *π*/2, the deformation takes place in the same direction as the direction of the magnetic field. **(D)** is a cartoon representation of the interaction between SNPs and lipids of the MLs. The intensity of the DC magnetic field was 0.09 *T* for the case of chain formation and 0.025 *T* for the ML deformation.

Through modification of the coprecipitation method, we prepared water-stable SNPs. Using transmission electron microscopy revealed that the nanoparticles have an average size of 12 *nm*. The measured hysteresis loop shows that nanoparticles exhibit superparamagnetic behavior. We have used solutions of SNP with different concentrations (1–5*%*) to prepare ML-containing SNP by the rehydration method. The experiments were performed in a homemade chamber, described in section 2.6 (Figure 3C.). We used the rehydrated ML solution with no further dilutions to fill the working zone of the chamber. An inverted light phase-contrast microscope (LEICA, DIL LED) with objectives of 20x, or 40x, is used to track the MLs. The experiments were filmed using a digital camera (LEICA) and recorded digitally. Figures 4A,C show optical microscopy images of a magnetoliposome obtained using 5*%* (A) and 1*%* (C) solutions, respectively. We observed a correlation between the concentration of MNPs and the formation of MLs. Higher concentrations of SNPs result in the formation of few MLs, and lower concentrations yield many MLs without SNP encapsulated. However, in the latter, we still observed a few MLs with SNP encapsulated. Within our protocol, we established that concentrations above the 5*%* inhibit the formation of liposomes. Thus, concentrations between 1 and 5*%* yield better results. No further treatment to the MLs was done; they were stored at room temperature and used immediately in the experiments with the DC magnetic field. With the designed magnetic chamber, we mapped the response of the MLs under an applied DC magnetic field. After a DC magnetic field is applied, the SNPs form chain-like arrangements inside the MLs; see from Figure 4A, the second figure in the line. If the direction of the magnetic field is rotated by $\frac{\pi }{2}$, the chains assemble in the same direction of the magnetic field, Figure 4A (third in the line). The chains fully disperse after the magnetic field is turned off. No deformation or poration over the liposome surface, when the chain formation takes place, was observed. SNPs inside the MLs line up in the direction of the DC magnetic field independent of the SNP concentration.

**FIGURE 5:**
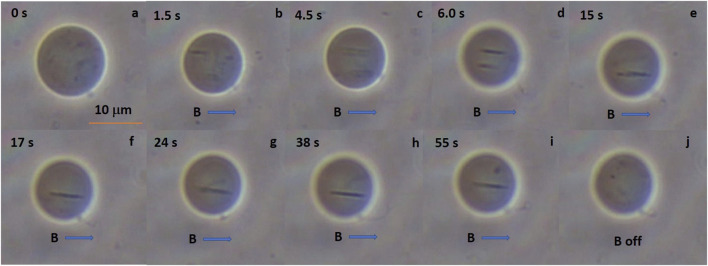
Chain formations inside MLs. When MLs are subjected to DC magnetic fields we can see that the small black dots (SNPs aggregates, 1*%* SNPs) form chains inside the MLs. **(A)** shows the MLs when no field is applied, the upper left number is the time counting in seconds. We can see the aggregates swirling inside the MLs. **(B)** is 1.5 s after the DC magnetic field is applied. A chain of SNPs is formed afterward, in **(C,D)** we can see two chain formations. After 15 s both chains collapse in one, in **(F)** the chain is stable displaying random movement **(G–I)**. Finally, in **J** the magnetic field is switched off and the chain is disassembled. We observe MLs for hours and the SNPs aggregates never leaves the MLs either with or without applied DC magnetic field.

Figure 5 shows a sequence of the chain formation over time. We observed the SNP aggregates moving randomly at the beginning of the experiment. The SNP aggregates and form large clusters. The SNP clusters are attracted to each other by the action of the magnetic field lines and repeal in the perpendicular direction of the DC magnetic field lines, forming the chains, see Figure 5B. Afterward, the chain assembled; we do not see any more changes. We observed chains of aggregated nanoparticles formed inside the MLs over a long period. If the magnetic field is switched off, the chain breaks apart, and SNPs aggregates move randomly again. In some experiments, MLs move due to a fluid flow; this can be due to magnetophoresis. We studied a total of 12 MLs at different concentrations.

When rehydration begins, MLs can encapsulate and accumulate SNP in the internal medium and over the membrane surface of the MLs, see Figures 4A,C, respectively. The second type of MLs is floppy and has an excess area. When a DC magnetic field is applied to the floppy MLs, they deform in the direction of the DC magnetic field, Figure 4C second in the line. The deformation type is prolate. When the direction of the magnetic field is rotated by $\frac{\pi }{2}$, the deformation is in the same direction as the applied field, Figure 4C third in the line. The cusp-like deformation might be due to the magnetic field strength due to pulling forces acting over the lipid membrane that is strong enough to squeeze the polar region of the MLs. The surface of the ML flattens to acquire cusp-like shapes. In this type of MLs, we do not observe chain formation, and the ML remains deformed as long as the magnetic field is applied. We do not find ML destruction or poration by increasing the strength of the magnetic field (up to the limit of our magnetic chamber). When the magnetic field is turned off, MLs return to their original fluctuating spheroidal state.

The SNP chain formation is explained in the base of SNP–SNP and SNP–membrane interactions. We are more interested in explaining ML deformation and leave pearl chain formation for future studies. The reason is that more precise knowledge of the encapsulated SNP concentration is needed to compare with a possible theory of pearl formation. The deformation of MLs can be understood if we analyze the density forces produced by the magnetic fields applied. In the next section, we develop a theory to explain prolate-shape deformations and describe the pearl-chain formation based on previous studies (Helfrich, 1973; Kummrow and Helfrich, 1991; Winterhalter and Helfrich, 1988; Yamamoto et al., 2010).

## 4 Theory

**FIGURE 6:**
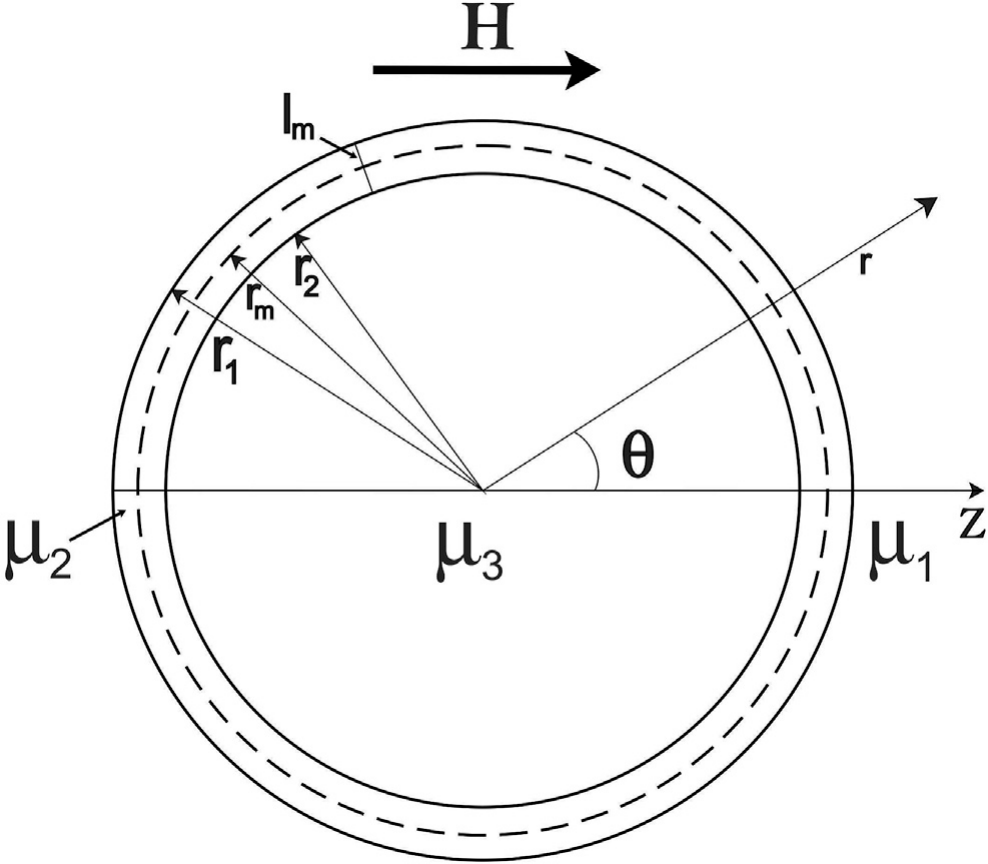
Model of a magnetoliposome in a DC magnetic field. MLs are immersed in a solution with permeability *μ*_1_. The direction of the DC is *z*, the interior of the MLs is filled with a material of magnetic permeability *μ*_3_ (*μ*_1_ = *μ*_3_), and the lipid membrane has magnetic permeability *μ*_2_. The width of the lipid membrane is *l*_*m*_, the radius of the outer and the inner membranes are *r*_1_ and *r*_2_, respectively, and the membrane radii are denoted by *r*_*m*_.

To discuss the shape deformation of MLs under the influence of an external magnetic field, we solved the relevant magnetostatic equations for the magnetic scalar potential. Details of the extended derivation of the model are in the Supporting Information. We modeled the magnetoliposome as a spherical thin layer shell where the three zones are identified *via* different magnetic permeability (see Figure 6). In this work, we solved the case where the inside and outside mediums of the magnetoliposome are the same. In the experimental section, vesicles form at some specific concentrations, and no further dilution is used to perform the experiments, then we can be sure that the solution is the same inside and outside the liposome. The general case, depicted in Figure 6, can be found elsewhere (de Lira Escobedo, 2015).

### 4.1 Theoretical Model

The magnetoliposome is modeled as a spherical thin layer shell (see Figure 6) where we defined three different regions with their magnetic permeability *μ*. For simplicity, inside and outside the lipid membrane is defined as a region with magnetic permeability $\mu_{1}\left(\mu_{1}=\mu_{3}\right).\;\mu_{2}$ defines a region of the space located inside the lipid membrane. The radius of the outer and the inner membranes are *r*_1_ and *r*_2_, respectively. The width of the lipid membrane *l*_*m*_ is defined as the subtraction of the outer and inner radii. The ratio between the deformation amplitude, defined as *s* (Winterhalter and Helfrich, 1988), and the membrane radii, defined as *r*_*m*_, characterizes the shape deformation of the magnetoliposome. Following the work of Yamamoto et al. (2010), the stable shapes acquired by a spherical liposome, when perturbed by an external field, are determined by minimizing the free energy of the system concerning the deformation parameter *s*. In this work, we obtained the work done by density forces over the membrane surface, when an external magnetic field is applied, in terms of the Maxwell stress tensor **T**.

We found the macroscopic magnetic field **H**, in all space, in terms of the magnetic scalar potential *φ* and considering membrane as an insulator shell. Its conductivity is much lower than that of water (hence **J** = 0), and the field distribution in the *z* direction has the form $H\left(r,\theta ,\phi \right)=-\nabla \varphi$. Furthermore, using the relation between the magnetic induction, the magnetic field *B* = *μH*, and the divergence equation ∇ ⋅ *B* = 0, we found that ∇^2^ *φ* = 0. Thus the potential *φ* satisfies the Laplace equation everywhere (Jackson, 1998). So far, the problem reduces to find the proper solutions in different regions to satisfy the relevant boundary conditions. The form of the potential in spherical coordinates is well known:

$$\varphi_{k}=\left(a_{k}r+\frac{b_{k}}{r^{2}}\right)\cos\theta ;k=1,2,3$$ (1)

where *k* = 1,2, and 3 represents the external, membrane, and inner medium, respectively, and the constants *a*’s and *b*’s are found using the boundary condition that fulfills the continuity of the field. Under an external magnetic field *H*_0_, the boundary conditions are such that **H**(*r* → *∞*) → *H*_0_ and is defined at *r* = 0. The continuity of the field is given by:

$$\frac{\partial \varphi_{j}}{\partial \theta }\left(r_{j}\right)=\frac{\partial \varphi_{j+1}}{\partial \theta }\left(r_{j}\right);j=1,2$$ (2)

where subscripts *j* represents the external (*j* = 1) and internal (*j* = 2) membrane surfaces.

$$\frac{\partial \varphi_{1}}{\partial r}\left(r_{1}\right)=\mu \frac{\partial \varphi_{2}}{\partial r}\left(r_{1}\right);$$ (3)

$$\mu \frac{\partial \varphi_{2}}{\partial r}\left(r_{2}\right)=\frac{\partial \varphi_{3}}{\partial r}\left(r_{2}\right)$$ (4)

where, since we take *μ*_1_ = *μ*_3_, we define *μ* as:

$$\mu =\mu_{2}/\mu_{1}$$ (5)

Applying these boundary conditions and solving the simultaneous equations, one can find the solution for constants *a*’s and *b*’s (see the Supporting Information) to define the magnetic scalar potential *φ*. Using **H**(*r*, *θ*, *ϕ*) = −∇*φ*, the magnetic field is written as:

$$H{\left(r,\theta \right)}_{k}=\left[\left(\frac{2b_{k}}{r^{3}}-a_{k}\right)\cos\theta \right]\hat{r}+\left[\left(\frac{b_{k}}{r^{3}}+a_{k}\right)\sin\theta \right]\hat{\theta }$$ (6)

The force densities over the membrane surface are generated when an external magnetic field is applied as a result of the discontinuity of the magnetic field due to the differences of the magnetic permeability in the interfaces surfaces and can be computed using the equation:

$$f_{j}=-n\cdot \left(T_{j+1}\left(r_{j},\theta \right)-T_{k}\left(r_{j},\theta \right)\right)j=1,2$$ (7)

where the dot symbol represents the product between a vector and a tensor, $n\equiv \hat{r}$ is the normal vector of the membrane surface, and **T** is the Maxwell stress tensor that physically represents the force per unit area (Jackson, 1998). To estimate the density forces, **f**_*j*_, we only take into account the magnetic term of the Maxwell stress tensor. The deformation of MLs is due to the normal and tangential components of the density force (see the expressions for *T*_*krr*_ and *T*_*krθ*_ in the Supporting Information). The forces given by Eq. 7 can deform the otherwise spheroidal MLs. The deformation can be in the direction of the DC magnetic field applied; this type of deformation is called prolate deformation. If the deformation is perpendicular to the DC magnetic field is called oblate deformation. To obtain the type and amplitude of deformations, we calculate the work done over the membrane surface by the DC magnetic field, this is given by

$$W_{mg}=\int f_{1}\cdot u\;dA_{1}+\int f_{2}\cdot u\;dA_{2}$$ (8)

where

$$\begin{array}{cc} f_{j}\cdot u & =\left\langle T_{jrr}-T_{\left(j+1\right)rr}\right\rangle u_{r}\\ & +\left\langle T_{jr\theta }-T_{\left(j+1\right)r\theta }\right\rangle u_{\theta }; \end{array}$$ (9)

$$dA_{j}=r_{j}^{2}\sin\theta d\theta d\phi ;$$ (10)

The components of the unit vector **u** that keeps the local area constant are (Winterhalter and Helfrich, 1988):

$$u_{r}=\frac{1}{2}s\left(3\cos^{2}\theta -1\right)$$ (11)

$$u_{\theta }=-s\cos\theta \sin\theta$$ (12)

where *s* (Winterhalter and Helfrich, 1988) is the deformation amplitude. For simplicity, we rewrite the expression for the magnetic field, defining parameters in terms of the radial and polar dependence (the problem poses azimuthal symmetry), and the constants obtained *via* the boundary conditions (see the Supporting Information) to obtain a simplified expression for the magnetic fieldwork as follows:

$$W_{mg}=\frac{8\pi }{15}sH_{0}^{2}\left\{r_{1}^{2}\Gamma_{1}+r_{2}^{2}\Gamma_{2}\right\}$$ (13)

where the dependence of the magnetic fieldwork with respect to the external DC magnetic field and the deformation amplitude is explicit. The Γ’s are the defined parameters and are addressed in the Supporting Information.

### 4.2 Free Energy of Magnetoliposomes Perturbed *via* DC Magnetic Fields

The energy contributions involved in the membrane deformation are the magnetic and the bending energies, and the stables shapes acquired by the MLs are due to a competition between these two energies in the form of the free energy (Yamamoto et al., 2010):

$$F=\Delta F_{be}-W_{mg}$$ (14)

where the Helfrich’s energy is express as (Helfrich, 1973):

$$\Delta F_{be}=\frac{48\pi }{5}\left(1-\frac{M_{sp}r_{m}}{6}\right)\kappa_{m}{\left(\frac{s}{r_{m}}\right)}^{2}$$ (15)

where *s* is the deformation amplitude, *κ*_*m*_ is the membrane bending rigidity, and *M*_*sp*_ spontaneous curvature. The stable shapes acquired by the MLs are determined by minimizing the free energy, Eq. (14), for the deformation parameter *s*. We obtain

$$\frac{s}{r_{m}}=\frac{r_{m}H_{0}^{2}}{36\left(1-\frac{M_{sp}r_{m}}{6}\right)\kappa_{m}}\left\{r_{1}^{2}\Gamma_{1}+r_{2}^{2}\Gamma_{2}\right\}$$ (16)

The ML attains a prolate-shape when *s* > 0, whereas if *s* < 0 the ML is of oblate shape. Eq. (16) can be simplified as follows

$$\begin{array}{cc} \frac{s}{r_{m}} & =\frac{r_{m}r_{1}^{2}\mu_{2}H_{0}^{2}}{6\Delta^{2}\left(1-\frac{M_{sp}r_{m}}{6}\right)\kappa_{m}}\left\{G_{0}+G_{1}\mu +G_{2}\mu^{2}\right.\\ & \left.+G_{3}\mu^{3}+G_{4}\frac{1}{\mu }\right\} \end{array}$$ (17)

where *G*’s terms are only functions of the vesicle geometry, the explicit form of Δ and *G*’s is in the Supporting Information.

**FIGURE 7:**
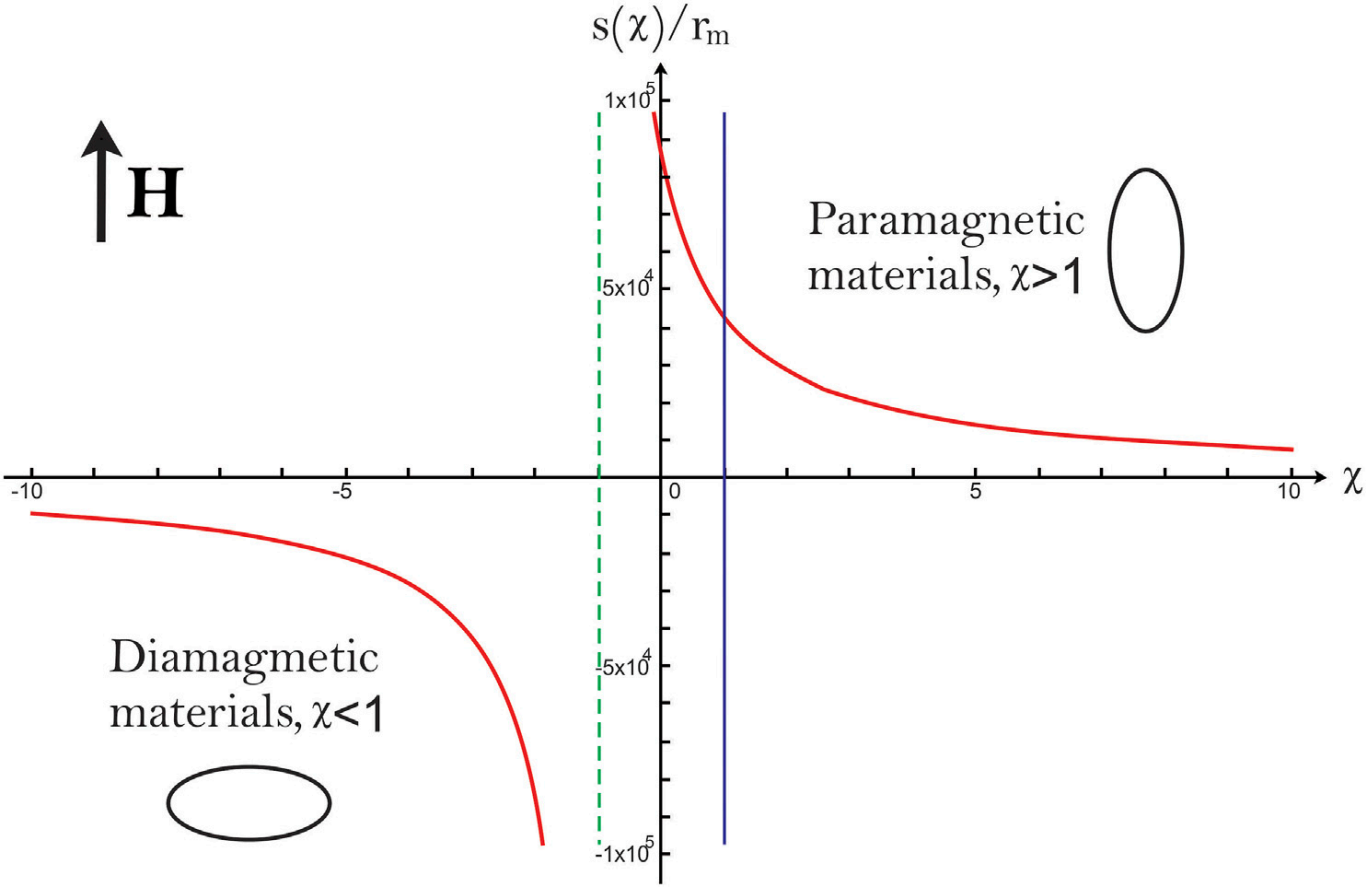
Morphological phase diagram of a MLs in a DC magnetic field. For *μ*_1_ = *μ*_3_ the MLs acquire a prolate shape when, the magnetic susceptibility, *χ* + 1 = *μ*_1_/*μ*_0_ = *μ*_3_/*μ*_0_ to a diamagnetic material the acquired shape is an oblate. For −1 < *χ* < 1 the MSL are prolates. The arrow indicates the direction of the magnetic field **H**; for symmetrical monolayer liposomes, we can assume that *c*_0_ = 0 and solutions for the quadratic expressions gives *μ*_1_ = 2*μ*_2_. For these values of permeability, the deformation can be either oblate or prolate; this line of coexistence is the green dotted line. The blue line is used to distinguish between paramagnetic and diamagnetic materials.

We can estimate the numerical values of the *G*’s in a practical case, for example, *r*_1_ = 10*μm* and *r*_2_ = *r*_1_ − *l* are:

$$\begin{array}{cc} G_{0} & =0.0143827\\ G_{1} & =-0.028764\\ G_{2} & =8.9856\\ G_{3} & =5.75539\times 10^{-6}\\ G_{4} & =1.43885\times 10^{-6} \end{array}$$ (18)

and *s* is obtained using reported values: (Kummrow and Helfrich, 1991; Loudet et al., 2010) *κ*_*m*_ = 1.9 × 10^−19^ J, *μ*_2_ = 1.221,451 × 10^–6^ and *H*_0_ = 1T. The behavior of *s* as function of the magnetic susceptibility *χ* + 1 = *μ*_1_/*μ*_0_ = *μ*_3_/*μ*_0_ being *μ*_0_ the magnetic susceptibility of vacuum, is shown in the Figure 7. From Eq. (17) we can see that a singular line can be obtained from the explicit form of Δ

$$\Delta =2c_{0}{\left(\mu -1\right)}^{2}-\left(2\mu +1\right)\left(\mu +2\right)$$ (19)

for symmetrical monolayer liposomes, we can assume that *c*_0_ = 0 and the solutions for the quadratic expressions give *μ*_1_ = 2*μ*_2_. By taking the last values of the permeability, the deformation can be either oblate or prolate. The line of coexistence is the green dotted line in Figure 7.

Only prolate deformations are obtained, when the medium inside and outside the liposomes are superparamagnetic solutions (Figure 7). The last is in agreement with our experimental results. The magnetic properties for the mediums influence the type of deformations we can obtain, and the deformation amplitude is proportional to the strength of the magnetic field applied.

## 5 Discussion

### 5.1 Superparamagnetic Nanoparticles Chain Formation

Several studies have shown that SNP self-organizes into chains under external magnetic fields, in bulk and confined systems (Myrovali et al., 2016; Andreu et al., 2011; Liu et al., 2015). This formation is reversible since the chains break up if the magnetic field is interrupted. In the literature, several groups have addressed the conditions and kinetics of the aggregation of superparamagnetic colloidal systems (Promislow et al., 1995; Martínez-Pedrero et al., 2007; Gajula et al., 2010). The driving force to form the aggregates is the interaction of the magnetic dipole induced in the nanoparticles by the external magnetic field.

Furthermore, studies in bulk and interface systems (Andreu et al., 2011; Zahn et al., 1997) have found that the equilibrium states depend only on a dimensionless magnetic coupling parameter ($\Gamma_{dd}=\frac{\mu_{0}\mu^{2}}{2\pi k_{B}T\sigma^{3}}$ measures the contact energy at *r* = *σ*, were *k*_*B*_ is the Boltzmann constant, *T* is the temperature, *r* is the aggregate radius, and *σ* is the aggregate diameter. This energy is related to the magnetic dipole–dipole of aggregates interaction and the packing fraction of the SNP (Andreu et al., 2011). SNP energy is minimal when they arrange a head to tail, and chain formation occurs.

When a liposome is not subject to a magnetic field, the SNP are a myriad of dots inside the vesicle. We use an optical microscope which allows inferring particle sizes in the hundreds of nanometers. The synthesis procedure allows dispersing SNPs in pure water, and the observation of these dots can be only SNPs aggregates.

The chain formation of these SNPs aggregates in the internal medium of MLs when a DC magnetic field is applied. No poration is detected, and chain formation vanishes once the DC magnetic field is switched off. SNPs attract to the magnetic field lines and repeal perpendicular to the direction of the field lines. The length and separation of the lines depend on the SNP concentration.

We show that SNPs aggregate in chains inside the MLs, and this can be a method to bring together biomolecules once the functionalization of SNPs for one specific task. Previous results demonstrate that embedded MNPs in small liposomes affect the bilayer structure stimulating drug release when an AC magnetic field is applied. These results suggest a potential use of MLs in biomedicine (Nappini et al., 2010).

### 5.2 Magnetoliposomes Deformations

In the rehydration procedure, besides MLs with encapsulated SNPs, MLs were observed without encapsulated SNPs and with an excess area. MLs without SNPs observed in the inner medium have a darker rim over the surface of the MLs, and when a DC magnetic field is applied, it deforms in the direction of the DC magnetic field.

The shape deformation of liposome under a magnetic field has been reported previously in Sandre et al. (2007), where iron oxide nanoparticles were coated with a citrate species to modify the pH of the system. However, in this study, we show that the response, expressed as a shape deformation of the liposomes, is mainly due to the SNP interaction of the encapsulated nanoparticles under the influence of an external magnetic field with the lipid membrane. Modifying the surface charge *via* a pH only leads to an increase in the onset of the external magnetic field for the deformation. Under the experimental conditions shown in this study, when the medium inside and outside the liposomes are superparamagnetic materials, the theory predicts only prolate deformations. The magnetic properties for the mediums influence the type of deformations we can obtain, and the deformation amplitude is proportional to the strength of the magnetic field applied.

## 6 Conclusion

MLs formed by rehydration produces two types of MLs. One that encapsulates SNPs in the internal medium and forms chains after a DC magnetic field is applied. The other type is floppy vesicles that deformed prolate after the magnetic field is applied. Both cases are reversible, and no damage is observed on the MLs surface. Here we propose to assemble SNP aggregates to form chains by using DC magnetic fields and afterward apply AC magnetic fields to increase the heat efficiency dissipation and enhance hyperthermia treatments. One of the outcomes of the theoretical approach is that, for the MLs deformation, the theory can reproduce the type of deformation by using the proper values for the main parameters reported (e.g., permeability, the strength of the magnetic field, bending stiffness, and radius of the vesicle). The model predicts only prolate deformations and considers only the case of symmetric conditions inside and outside the MLs. A generalization of the theoretical model can be made for *μ*_1_ ≠ *μ*_3_ to explore if it is possible to predict different shape deformations. The theoretical model foresees that MLs, in paramagnetic medium, will deform prolate, and in the diamagnetic medium will deform oblate. Cryo-TEM studies can confirm the presence of SNPs either in the surface or the inner medium of liposomes.

## Data Availability

The original contributions presented in the study are included in the article/Supplementary Material; further inquiries can be directed to the corresponding authors.
