# Supplementary figures and images for: Effects of DC Magnetic Fields on Magnetoliposomes
Source: Front Mol Biosci. 2021 Sep 13;8:703417. doi: 10.3389/fmolb.2021.703417 (PMC8473709; doi:10.3389/fmolb.2021.703417)

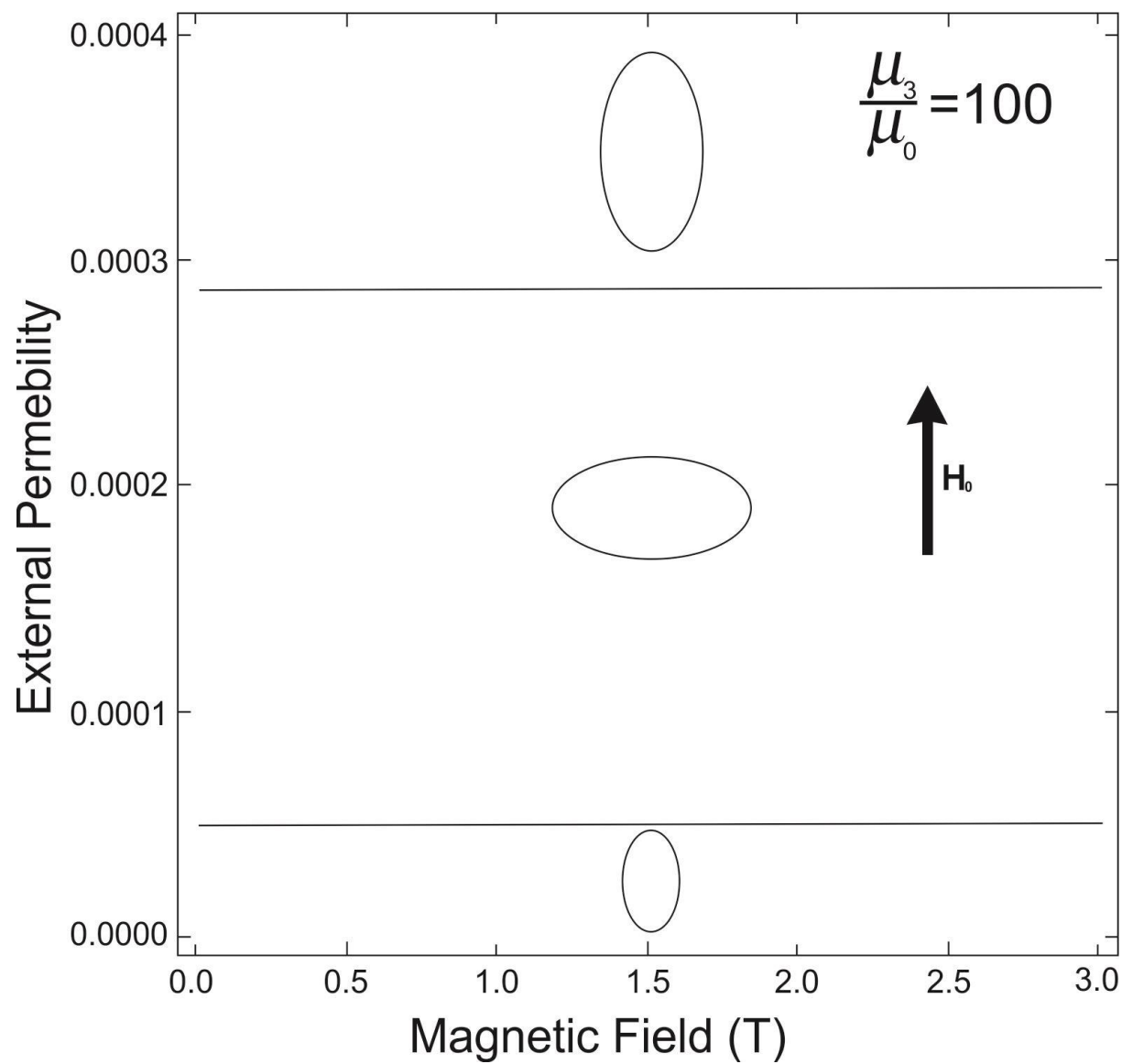

Supplement: Supplementary file 1 [file DataSheet2.PDF]

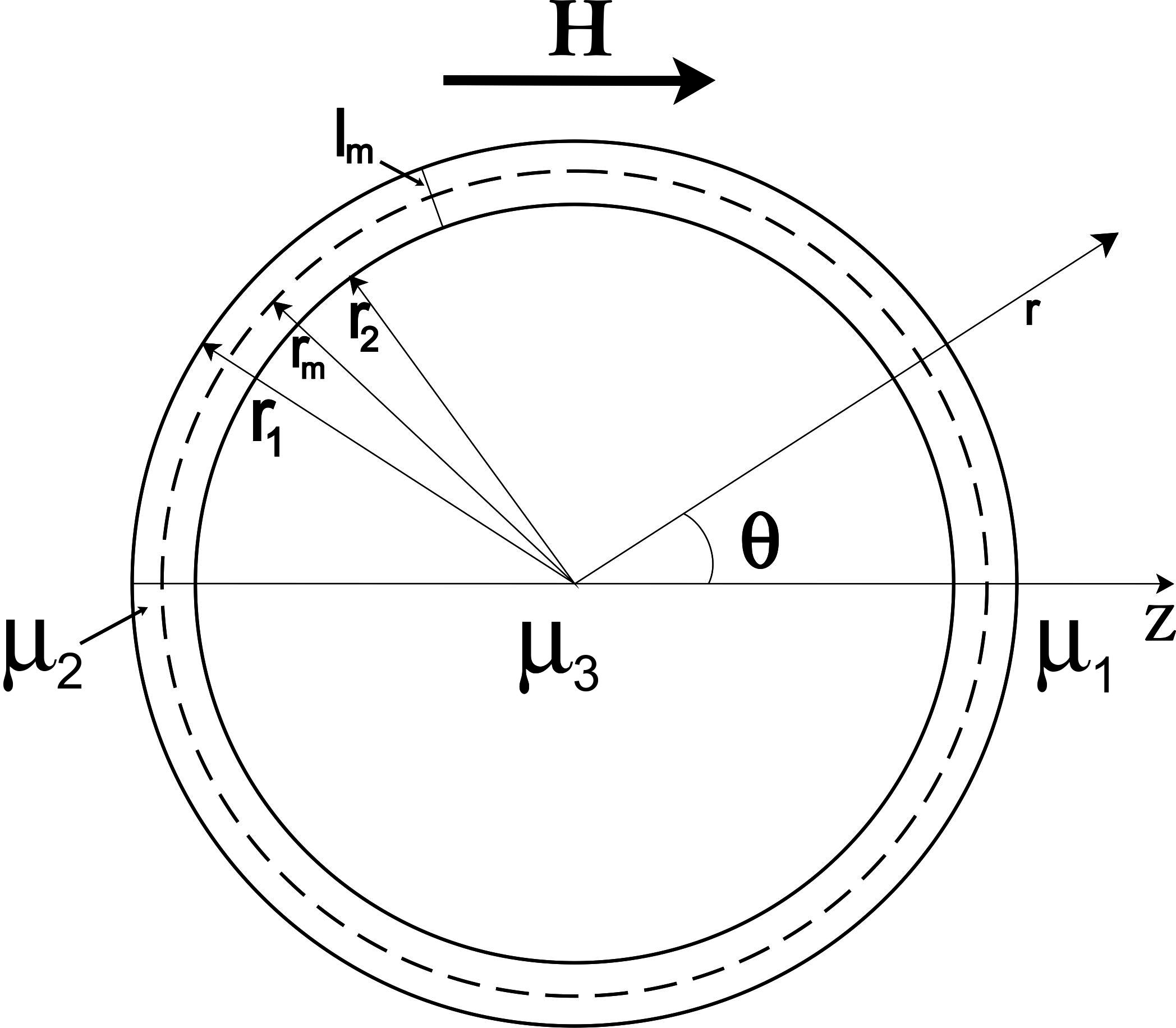

Supplement: Supplementary file 2 [file Image3.JPEG]

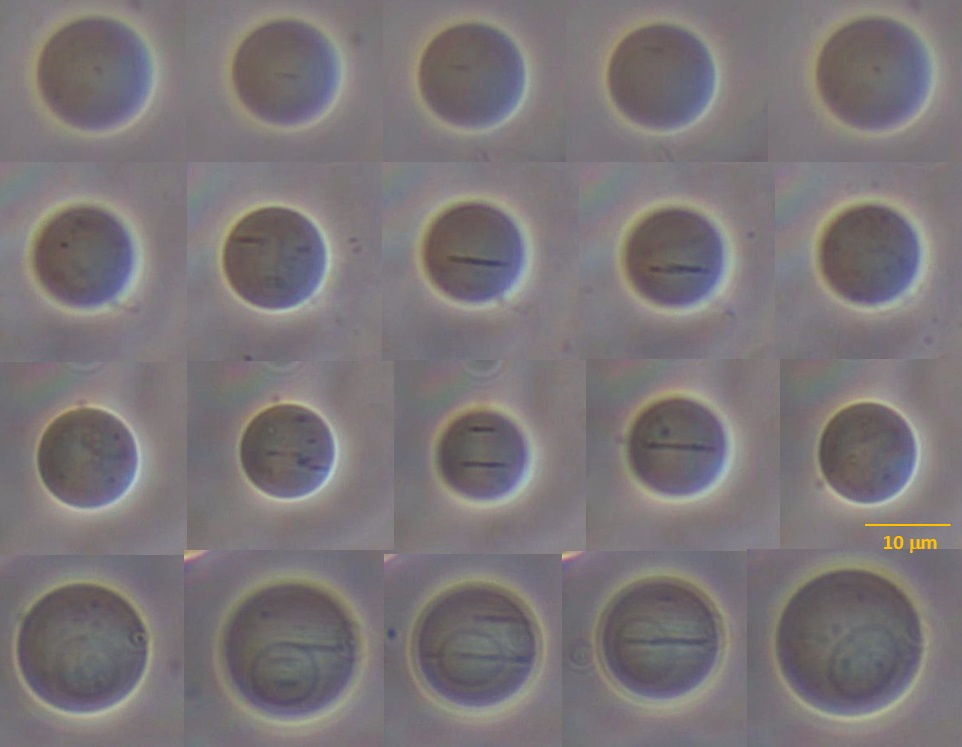

Supplement: Supplementary file 4 [file Image4.JPEG]

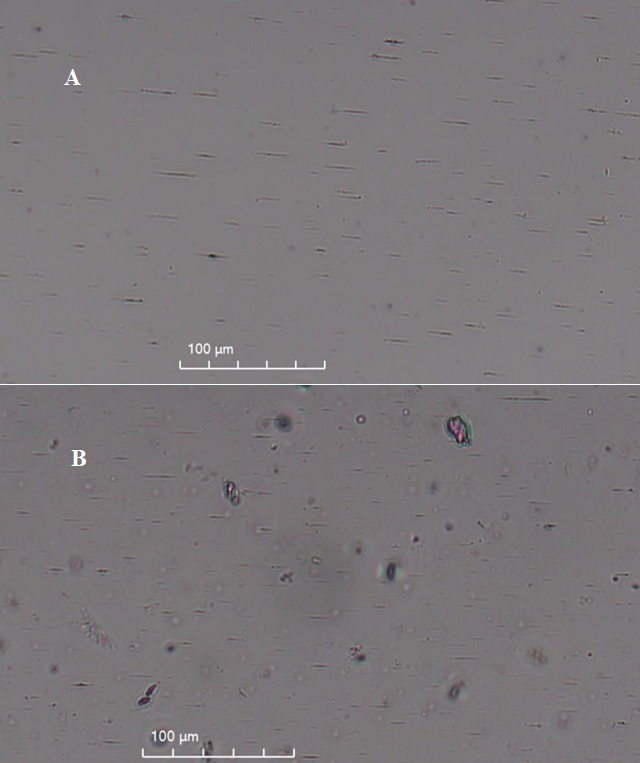

Supplement: Supplementary file 5 [file Image2.JPEG]
