# Supplementary material for: Effects of DC Magnetic Fields on Magnetoliposomes
Source: Front Mol Biosci. 2021 Sep 13;8:703417. doi: 10.3389/fmolb.2021.703417 (PMC8473709; doi:10.3389/fmolb.2021.703417)
Supplement: Supplementary file 6 [file DataSheet1.pdf]

Supplementary Material

1 SUPPLEMENTARY TABLES AND FIGURES

2 EXPERIMENTAL CHARACTERIZATION

2.1 X-ray diffraction Analysis.

XRD description: X-ray diffractometer Smartlab Rigaku (Cu Kα X-ray tube; 40 Kv, 44 mA) was used for this work). Measurement conditions: 2q range: 5 to 100 Step: 0.02 Speed scan: 1 sec.

Figure S1 shows the diffractogram of iron oxide nanoparticles synthesized (top). According to peak matching with data from the PDF4 of the ICDD, conventional X-ray diffraction analysis consistently shows that the sample is composed of two phases: magnetite and maghemite. As can be seen, these two phases explain the diffraction pattern of the Iron Oxide Nanoparticles. According to the RIR Quantitative Analysis (calculated automatically by XPert HighScore software), the percentage of magnetite is approximately 63$\%$ and for maghemite 37$\%$.

2.2 SNP in suspension under DC magnetic Fields

Control experiment to show that SNP align in the direction of the magnetic field without liposomes. We put 6.8 μl of suspended SNP in pure water at 1$\%$ and 3$\%$ dilution. Figure S2 shows a top view of the chamber experiment where the solutions of diluted SNP. After applying a DC magnetic field of 0.09 T, we observe the formation of linear chains in both concentrations.

2.3 Chain formation inside MLS

THEORY

In order to discuss the shape deformation of the magnetoliposomes under the influence of an external magnetic field, we solved the relevant magnetostatic equations for the magnetic scalar potential. Herewith we present details of the extended derivation of the model. The magnetoliposome is modeled as a spherical thin layer shell where the three zones specifically are identified via different magnetic permeability (see Figure S3). We present a particular case where the inside and outside mediums of the magnetoliposome are constructed with the same material. The general case depicted in Figure S3 can be found elsewhere **?**.

MLS is modeled as a spherical thin layer shell of permeability μ_2 placed in a region of the space with permeability μ_1. The width of the lipid membrane is l_m, the radius of the outer and the inner membranes are r_1 and r_2 respectively, and the membrane radii are denoted by r_m, see Figure S3. We find the field distribution in all space due to a DC magnetic field in the z direction from a scalar potential $\mathbf{H}(r, \theta, \phi) = -\nabla\varphi$. The membrane is considered a lossy dielectric medium and $\mathbf{J} = 0$. Furthermore, using the relation between the magnetic induction and the magnetic field $B = \mu H$ and the divergence equation $\nabla \cdot B = 0$, we find that $\nabla^2\varphi = 0$, thus the potential ϕ satisfies the Laplace equation everywhere **?**.

The form of the potential in spherical coordinates is well known:

$$\varphi_k = \left(a_k r + \frac{b_k}{r^2}\right) cos\theta; \quad k = 1, 2, 3 \tag{S1}$$

where k=1,2 and 3 represents the external, membrane and inner medium respectively and the constants a's and b's are found using the boundary condition that fulfills the continuity of the field. Under an external magnetic field H_0 the boundary conditions are such that $\mathbf{H}(r \to \infty) \to H_0$ and is defined at $r = 0$. The

continuity of the field is given by:

$$\frac{\partial \varphi_j}{\partial \theta}(r_j) = \frac{\partial \varphi_{j+1}}{\partial \theta}(r_j); \quad j = 1, 2 \tag{S2}$$

where subscripts j represent the external (j=1) and internal (j=2) membrane surfaces.

$$\frac{\partial \varphi_1}{\partial r}(r_1) = \mu \frac{\partial \varphi_2}{\partial r}(r_1); \tag{S3}$$

$$\mu \frac{\partial \varphi_2}{\partial r}(r_2) = \frac{\partial \varphi_3}{\partial r}(r_2) \tag{S4}$$

where, since $\mu_1 = \mu_3$, we define μ as

$$\mu = \mu_2/\mu_1 \tag{S5}$$

The solution for constants a's and b's are:

$$a_1 = -H_0 \tag{S6}$$

$$a_2 = \left[\frac{3(2\mu + 1)}{2\frac{r_2^3}{r_1^3}(\mu - 1)^2 - (2\mu + 1)(\mu + 2)} \right] H_0 \tag{S7}$$

$$a_3 = \left[\frac{9\mu}{2\frac{r_2^3}{r_1^3}(\mu - 1)^2 - (2\mu + 1)(\mu + 2)} \right] H_0 \tag{S8}$$

$$b_1 = \left[-\frac{(2\mu + 1)(\mu - 1)}{2\frac{r_2^3}{r_1^3}(\mu - 1)^2 - (2\mu + 1)(\mu + 2)} \right] (r_1^3 - r_2^3) H_0 \tag{S9}$$

$$b_2 = \left[\frac{3(\mu - 1)}{2\frac{r_2^3}{r_1^3}(\mu - 1)^2 - (2\mu + 1)(\mu + 2)} \right] r_2^3 H_0 \tag{S10}$$

$$b_3 = 0 \tag{S11}$$

a_3 is related to the internal magnetic field and when $\mu_2 \gg \mu_1$ and $\mu_2 \ll \mu_1$ there will be a magnetic shielding *i.e* there is a significant decrease on the internal magnetic field. Using $\mathbf{H}(r, \theta, \phi) = -\nabla \varphi$, the magnetic field is written in the next form:

$$\mathbf{H}(r,\theta)_k = \left[\left(\frac{2b_k}{r^3} - a_k\right) cos\theta\right]\hat{\mathbf{r}} + \left[\left(\frac{b_k}{r^3} + a_k\right) sin\theta\right]\hat{\theta} \tag{S12}$$

Densities forces over the membrane surface are generated when a magnetic field is applied, this densities forces are the result of the discontinuity of the field due to the differences of the magnetic permeability in the surfaces interfaces and can be computed using the equation (we follow a similar procedure reported by Yamamoto $etal.$ **?**, where density forces over a spherical liposome under an AC electric field are calculated to study vesicle deformations).

$$\mathbf{f}_j = -\mathbf{n}\cdot\Big(T_{j+1}(r_i,\theta) - T_i(r_j,\theta)\Big) \tag{S13}$$

where the dot represents the product between a vector and a tensor, $\mathbf{n} \equiv \hat{\mathbf{r}}$ is the normal vector of the membrane surface and T is the Maxwell Stress Tensor that physically represents the force per unit area it is defined by **?**:

$$\begin{aligned} T_{kij} &= \varepsilon_k\left(E_{ki}E_{kj} - \frac{1}{2}\delta_{ij}E_k^2\right) \\ &+ \frac{1}{\mu_k}\left(B_{ki}B_{kj} - \frac{1}{2}\delta_{ij}B_k^2\right); \quad k = 1,2,3 \end{aligned} \tag{S14}$$

in this definition i and j are dummy subscripts. To estimate the density forces, f_j, we only take into account the magnetic term of the equation S14. The deformation of MLS are due to the normal and tangential components of the density force; the stress tensor components take the form:

$$T_{krr} = \frac{\mu_k}{2}\left(|H_{kr}|^2 - |H_{k\theta}|^2\right); \quad k = 1,2,3 \tag{S15}$$

and

$$T_{kr\theta} = \mu_k H_{kr}H_{k\theta}; \quad k = 1,2,3 \tag{S16}$$

The forces given by the equation S13 can deform the otherwise spheroidal MLS. The deformation can be in the direction of the DC magnetic field applied, this type of deformation is called prolate deformation. If the deformation is perpendicular to the DC magnetic field is called oblate deformation. To obtain the type and strength of deformations we calculate the work done over the membrane surface by the DC magnetic field, this is given by

$$W_{mg} = \int \mathbf{f}_1\cdot\mathbf{u}\,dA_1 + \int \mathbf{f}_2\cdot\mathbf{u}\,dA_2 \tag{S17}$$

where:

$$\mathbf{f}_j \cdot \mathbf{u} = \langle T_{jrr} - T_{(j+1)rr} \rangle u_r + \langle T_{jr\theta} - T_{(j+1)r\theta} \rangle u_\theta; \tag{S18}$$

$$dA_j = r_j^2 sen\theta d\theta d\phi; \tag{S19}$$

The components of the unit vector $\mathbf{u}$, that keeps the local area constant are **?** :

$$u_r = \frac{1}{2} s \left(3cos^2\theta - 1\right) \tag{S20}$$

$$u_\theta = -scos\theta sin\theta \tag{S21}$$

where s is the deformation amplitude. For simplicity we can write the magnetic field as

$$H(r,\theta)_k = \tau(r)_k cos\theta \hat{\mathbf{r}} + \eta(r)_k sin\theta \hat{\theta}; \quad k = 1,..,3 \tag{S22}$$

where

$$\tau(r)_k = \frac{2b_k}{r^3} - a_k; \quad k = 1,2,3 \tag{S23}$$

and

$$\eta(r)_k = \frac{b_k}{r^3} + a_k; \quad k = 1,2,3 \tag{S24}$$

we obtain the next expression for the magnetic field work

$$W_{mg} = \frac{8\pi}{15} s H_0^2 \left\{ r_1^2 \Gamma_1 + r_2^2 \Gamma_2^m \right\} \tag{S25}$$

where

$$\begin{aligned} \Gamma_k = {} & \varepsilon_k \left[|\tau_k(r_k)|^2 + |\eta_k(r_k)|^2 \right] \\ & - \varepsilon_{k+1} \left[|\tau_{k+1}(r_k)|^2 + |\eta_{k+1}(r_k)|^2 \right] \\ & + \varepsilon_{k+1} \left[\tau_{k+1}(r_k) \eta_{k+1}(r_k) \right] - \varepsilon_k \left[\tau_k(r_k) \eta_k(r_k) \right] \end{aligned} \tag{S26}$$

Free Energy of magnetoliposomes in DC Magnetic Fields. The energies contributions that are involved in the membrane deformation are the magnetic and the bending energy, and the stables shapes acquired by the MLS is due to a competition between this two energies in the form of free energy **?**:

$$F = \Delta F_{be} - W_{mg} \tag{S27}$$

where:

$$\Delta F_{be} = \frac{48\pi}{5}\left(1 - \frac{M_{sp}r_m}{6}\right)\kappa_m\left(\frac{s}{r_m}\right)^2 \tag{S28}$$

where s is the deformation amplitude, κ_m is the membrane bending rigidity and M_{sp} spontaneous curvature. The stables shapes acquired by the MLS are determined by minimizing the free energy respect to the parameter of the deformation, s. We obtain

$$\frac{s}{r_m} = \frac{r_m H_0^2}{36\left(1 - \frac{M_{sp}r_m}{6}\right)\kappa_m}\left\{r_1^2\Gamma_1 + r_2^2\Gamma_2\right\} \tag{S29}$$

The MLS attains prolate-shape when $s > 0$ whereas if $s < 0$ the MLS is oblate-shape. Equation S29 can be simplified as follows

$$\begin{aligned}\frac{s}{r_m} = \frac{r_m r_1^2 \mu_2 H_0^2}{6\Delta^2\left(1 - \frac{M_{sp}r_m}{6}\right)\kappa_m}&\Big\{G_0 + G_1\mu + G_2\mu^2 \\ &+ G_3\mu^3 + G_4\frac{1}{\mu}\Big\}\end{aligned} \tag{S30}$$

where the G's terms are only functions of the vesicle geometry, the explicit form of Δ and G's are:

$$\begin{aligned}
\Delta &= 2c_0(\mu-1)^2 - (2\mu+1)(\mu+2) \\
G_0 &= 2(1-c_0^3)(2+c_0^3) - (1+2c_0^3)^2 - (1-c_0^3)^2 + 9c_0^2 \\
G_1 &= (1+2c_0^3)^2 + (2+c_0^3)^2 \\
&\quad - 4(1+2c_0^3)(1-c_0^3) - 2(1-c_0^3)(2+c_0^3) - 18c_0^2 \\
G_2 &= (1+2c_0^3)^2 - 4(1-c_0^3)^2 - (2+c_0^3)^2 + 9c_0^2 \\
G_3 &= 4(1-c_0^3)^2 \\
G_4 &= (1-c_0^3)^2 \\
c_0 &= \frac{r_2}{r_1}
\end{aligned} \tag{S31}$$

2.4 Figures

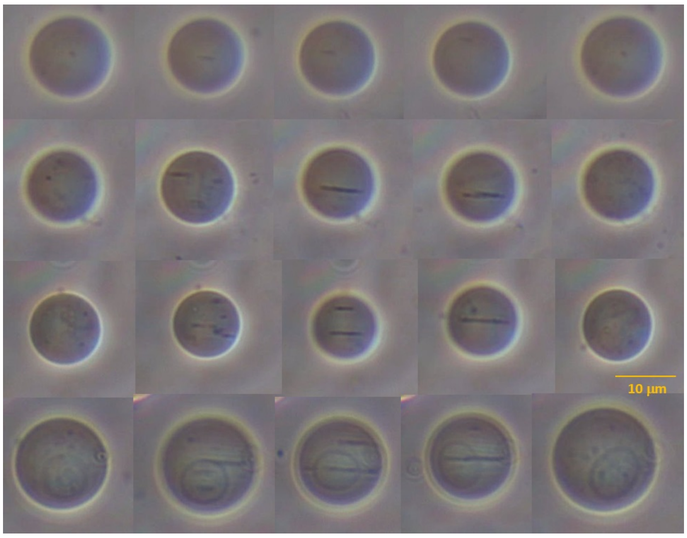

Figure S1. Chain formations inside MLS*When MLS are subject to DC magnetic field, we can see that the small black dots (SNPs aggregates, 1 % SNP first row, and second to fourth 3 %) form chains inside the MLS. The last row shows a multilamellar vesicle.*

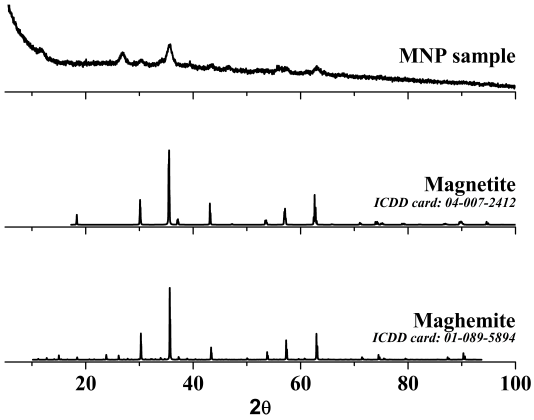

Figure S2. XRD diffractograms. *X-ray diffractograms of Iron Oxide nanoparticles sample revealing experimental peaks that match with simulated magnetite and maghemite according to PDF4 of the ICDD database.*

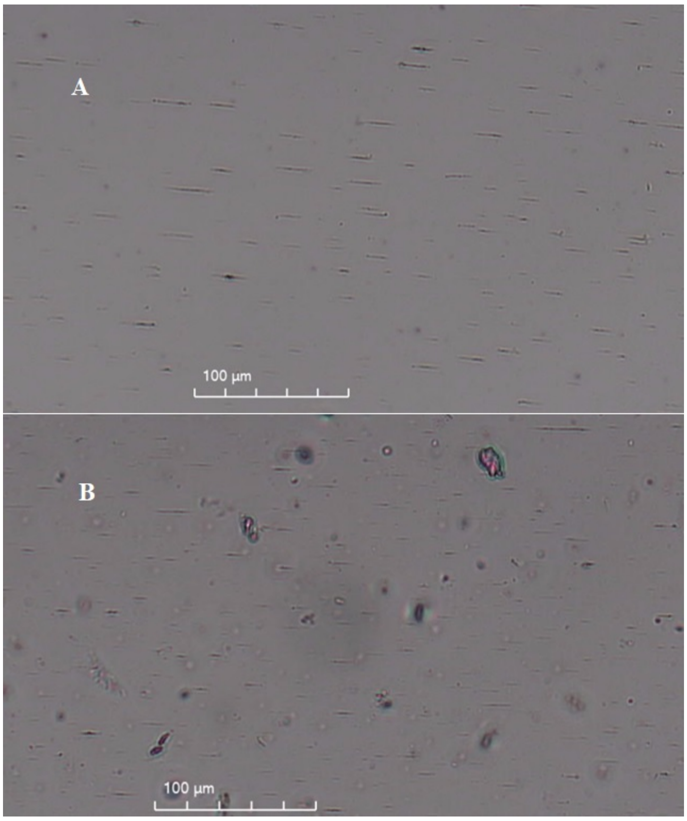

Figure S3. Linear chain formation of bulk SNPs. *Objective 10x. Light Field Rectangular Linear Microfluidic Chamber. A. Chain formation along DC Magnetic Field. 1% SNPs concentration. B. Chain formation along DC Magnetic Field. 3% SNPs concentration.*

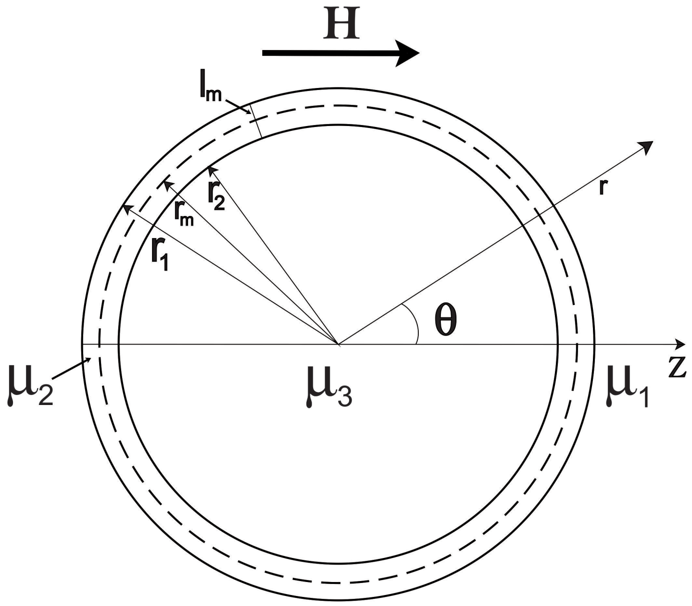

Figure S4. Model of a magnetoliposome in a DC magnetic field. MLS is immersed in a solution with permeability μ_1. The direction of the DC is z, the interior of the MLS is filled with a material of magnetic permeability μ_1, the lipid membrane has magnetic permeability μ_2. The width of the lipid membrane is l_m, the radius of the outer and the inner membranes are r_1 and r_2 respectively, and the membrane radii are denoted by r_m.
